# Using the Centers for Disease Control and Prevention’s Stay Independent Checklist to Engage a Community of American Indians and Raise Awareness About Risk of Falls, 2016

**DOI:** 10.5888/pcd14.160395

**Published:** 2017-01-19

**Authors:** Janet Popp, Debra L. Waters, Karen Leekity, Donica Ghahate, Jeanette Bobelu, Ross Tsikewa, Carla J. Herman, Vallabh Shah

**Affiliations:** 1 School of Medicine, University of New Mexico Health Sciences Center, Albuquerque, New Mexico; 2 Dunedin School of Medicine and School of Physiotherapy, University of Otago, Dunedin, New Zealand; 3 Zuni Wellness Center, Zuni Pueblo, New Mexico.

## Abstract

**Background:**

The unintentional death rate from falls is higher among American Indians from the US Southwest than from other regions in the country. The Zuni Pueblo is a geographically isolated, rural American Indian community located in western New Mexico. Education and screening for falls risk is lacking in this community and may be needed to reduce falls and falls-related illness and death.

**Community Context:**

Building on a 17-year relationship with the Zuni Health Initiative, meetings were held with Zuni tribal leadership, staff from the Zuni Senior Center and Zuni Home Health Services, members of the Zuni Comprehensive Community Health Center, Indian Health Service, and Zuni community health representatives (CHRs) to discuss elder falls in the community. Existing infrastructure, including CHRs who were already trained and certified in diabetes education and prevention, provided support for the study.

**Methods:**

Tribal leadership agreed that CHRs would be trained to administer the Centers for Disease Control and Prevention’s (CDC’s) Stay Independent checklist to assess falls risk. They administered the checklist during one-on-one interviews in Shiwi (Zuni native language), English, or both to a convenience sample of 50 Zuni elders.

**Outcomes:**

Mean age of participants was 72 (standard deviation, 7.4) years, and 78% were women. Fifty-two percent reported at least 1 fall during the past year; 66% scored 4 or more on the CDC Stay Independent checklist, indicating elevated risk for falls. CHRs reported that the checklist was easy to administer and culturally accepted by the elder participants.

**Interpretation:**

This study broadened the Zuni Health Initiative to include falls risk screening. Self-reported falls were common in this small sample, and the incidence was significantly higher than the national rate. These results highlight the need for community engagement, using culturally acceptable falls screening, to promote falls education and implement falls prevention programs.

## Background

Native elders are essential to preserving the culture and history of tribal communities, and falls-related injuries can put elders’ presence in their communities at risk. One-third of US community-based adults aged 65 years or older fall each year (1,2), and the unintentional fall death rate is higher in American Indians (AIs) from the US Southwest than from other regions in the country (3). In 2000, the US age-adjusted falls-related crude death rate was 1.4 times higher for AIs and Alaska Natives (ANs) than for the non-Native population (3). A National Resource Center on Native American Aging survey of 18,000 tribal elders aged 55 years or older reported that 40% of women and 34% of men had 1 or more falls during the previous year (4). In New Mexico, falls are the leading cause of injury-related deaths, hospitalizations, and emergency department visits for adults aged 65 years or older (5).

Falls can be particularly serious for older AIs who experiences more comorbidity and chronic illnesses, particular diabetes and arthritis, than does the general population (6,7,8,9). Zuni Indian Health Service (IHS) surveillance reported that Zuni elders have high rates of type 2 diabetes (59% in adults aged ≥60 years) and chronic kidney disease (48% in adults aged ≥60 years).

The Zuni community has a long-standing relationship with the University of New Mexico Health Sciences Center (HSC) that has focused on prevention and treatment of diabetes and chronic kidney disease; to date, however, this partnership has not addressed elder falls. For this study, we used established community-based participatory research practices in the Zuni community and collaborated with community health representatives (CHRs) to review the Centers for Disease Control and Prevention’s (CDC’s) Stay Independent checklist for cultural sensitivity as the first step to build relationships for falls prevention. This collaboration allowed for falls screening and brief falls prevention education in collaboration with the Zuni community.

## Community Context

Zuni Pueblo is home to a small Native American tribe located in rural New Mexico. This socioeconomically disadvantaged population faces a major public health challenge from growing health disparities, and members of Zuni Pueblo live in geographic isolation with limited access to rehabilitative and supportive services. If a Zuni elder sustains a fall-related injury, the closest tribal assisted living or skilled nursing facility is 100 miles from the reservation. The Zuni tribe has no public transportation system; however, Zuni IHS and other health programs, including the Zuni Health Initiative, assist people with transportation. There is an intermittent van service in Zuni, but regular service is unavailable (10). Home health physical therapy services, often needed to recover from a serious fall-related injury, are unavailable at the Pueblo. Therefore, Zuni elders may be forced to choose between leaving their community and social network to obtain intensive rehabilitative services or remain in the community with unmet needs and increased risk of not regaining their prior level of function. These combined factors highlight the need for falls-prevention strategies as a primary prevention intervention to prevent injurious falls and preserve aging in place in this community.

The HSC has a 17-year research partnership with the Zuni tribe, which started in 1997 when the Zuni tribal governor contacted HSC about the sudden increase in renal failure. In response, HSC established the original Zuni Kidney Project (1998–1999) using a community-based participatory research model and 3 subsequent National Institutes of Health R01 grants to study renal disease and comorbidities. In 2009, the Zuni Kidney Project activities were extended, and the Zuni Health Initiative was created to address tribal leadership concerns about increasing rates of chronic diseases. The Zuni Health Initiative educational program has identified barriers to health care, evaluated knowledge and perception of diabetes, measured health literacy, and assessed patient activity in chronic disease self-management in the Zuni community (11–17).

Through this partnership, strong working relationships between the Zuni Tribal Council, HSC, and the Zuni IHS have been established. A small number of Zuni Community Health Representatives (CHRs) have also been trained and certified as health educators. The tribal leadership of Zuni Pueblo recognized that it must develop innovative methods to implement proven interventions to prevent injury and promote aging in place for tribal members, and that was the starting point for this collaborative study.

## Methods

In March 2015, the senior center director (K.L.) spoke to the principal investigator (V.S.) of the Zuni Health Initiative and presented an overview of elder services to his staff so that they could understand the issues and challenges elders face as they age in place at Zuni Pueblo. During the presentation, the concern about falls and falls-related injuries was expressed. In particular, the senior center director (K.L.) noted a lack of programming for homebound elders; it was also noted that many Zuni elders require or prefer home-based services and specifically that falls-risk interventions were not available. The principal investigator approached the tribal leadership to ask if they were interested in discussing falls prevention and falls prevention research with some of the research team. In July 2015, a meeting was subsequently arranged with tribal leadership, Zuni leadership (including the governor, council members, and Zuni IHS providers) and Zuni Senior Center (K.L.), CHRs, and representatives from the Zuni IHS. National-level data on falls risk in AI communities were presented. A video was shown (D.W.) on a community-based falls-prevention program that uses the exercises from the CDC compendium–approved program Otago Exercise Program that was developed in New Zealand (18). Consequently, the governor gave support and approval for this study.

Permission was requested and received from the tribal council to train the CHRs to assess a screening instrument (CDC STEADI resource, Stay Independent self-assessment tool) and collect preliminary data on falls risk. The self-assessment tool was chosen, because it is a consumer handout with simple strategies to reduce falls risk such as an annual medication review and vision examination, removing home trip hazards, and increasing physical activity. In addition, Stay Independent offers a simple checklist that can be completed by an older adult or administered by another person.

To confirm the cultural appropriateness of the Stay Independent resource as well as the self-assessment tool, a focus group with 5 Zuni CHRs was held at HSC in August 2015. This meeting was to ensure that all possible phrasing or statements considered I:ba’naye (“taboo” in Shiwi, the Zuni native language) were not used. Such phrasing could result in a fatalistic perception that specific content is a bad omen, ensuring that elders will put themselves at risk of falling and that participation in the study thus would be dangerous (important note: Shiwi is not a written language). The Zuni Health Initiative CHRs speak both English and Shiwi and have 15 years of experience delivering educational home-based interventions for chronic conditions. Therefore, the CHRs were confident in administering the tool in Shiwi.

The HSC research team developed a series of focus group questions. The CHR-led focus group questions relevant to Stay Independent materials were 1) What do you think about this set of questions?, 2) Do you think there are any questions that are not culturally appropriate?, 3) Do you think Zuni elders would be willing to answer questions about falls?, 4) How long do you think it would take to conduct each set of questions with a Zuni elder?, 5) Would it be possible to use this document in a group discussion?, and 6) Do elders or family members talk to you about falls during home visits? The Zuni CHRs who attended the focus group are trained and certified health educators for a diabetes project at Zuni. The CHRs confirmed that falls are a common issue of concern during home visits for the diabetes project. All agreed that the Stay Independent questions were culturally appropriate and anticipated that elders would be willing to provide answers. The CHRs recommended that one-on-one interviews would be more effective in engaging elders than group discussion of the resource checklist and anticipated these interviews would take less than 20 minutes. The CHRs also recommended offering a small compensation ($25 gift cards) for study participation to show appreciation for elders’ contribution to the study. CHRs conducted the Stay Independent checklist interviews in Shiwi, English, or both according to elder preferences. CHRs collected and entered the data for analysis by the HSC team (J.P., D.W.).

To promote and recruit for the study, CHRs designed and posted flyers at the Zuni Senior Center. During the senior center’s routine schedule of lunch and afternoon activities, CHRs made 3 visits to engage elders in one-on-one conversations to explain the survey and its purpose to understand fall risk at Zuni Pueblo and to collect data from the self-assessment tool. The director of elderly services at the senior center maintained a sign-up list.

This study was approved by the HSC’s Human Research Review Committee and the Zuni IHS institutional review board (no. 10–249). All participants provided written informed consent.

## Outcomes

This study accomplished 3 objectives: 1) broaden the scope of the Zuni Health Initiative to work with CHRs to assess the CDC STEADI resources for cultural appropriateness and to administer the screening tool to understand common fall risk factors among Zuni elders aged 60 years or older; 2) engage the community to consider modifiable fall risk factors and consider next steps to empower elders to participate in fall prevention; and 3) share and discuss study results with Zuni tribal leadership to inform future planning for policies and funding proposal development.

Fifty-six elders signed up at the Zuni Senior Center, and 50 were selected on the basis of availability. Data on general health (diagnosis of type 2 diabetes or hypertension, history of heart attack or stroke, or on dialysis) and demographics (age and sex) were obtained (Table 1). Study participants reported high incidence of chronic health conditions. Of the participants, 78% were female and had a mean age of 72 (standard deviation, 7.4) years, 65% had been diagnosed with type 2 diabetes, 73% had hypertension, and 6% were on dialysis.

**Table 1:** Demographic Characteristics of Participants (N = 50), Zuni Health Initiative, New Mexico, 2015

Characteristic	Value^a^
**Sex**	
Female	39 (78)
Male	11 (22)
**Mean (standard deviation) age, y**	72 (7.4)
**Health status**	
Type 2 diabetes	31 (65)
Hypertension	36 (73)
History of heart attack	2 (4)
History of stroke	5 (10)
On dialysis	3 (6)

a Values presented as no. (%), unless otherwise indicated. Values may not sum to total because of missing data.

To achieve the first objective, after receiving tribal council and ethical approval of the Stay Independent checklist, the CHRs administered it and provided introductory falls prevention education. The CHRs were successful in this first step in that the tool was administered to all participants, and all checklists had complete information. All CHRs reported the participants having no trouble understanding the questions; inter-rater reliability was not assessed.

The results of the Stay Independent assessment demonstrated the need for falls prevention in the Zuni community (Table 2). CDC reports that a Stay Independent checklist score of 4 points or higher indicates elevated fall risk; 66% of study participants scored 4 or more points, and 52% reported a fall during the past year or sometimes feeling unsteady when walking. Seventy-one percent indicated that they are worried about falling, a risk factor that can lead to activity restriction and further loss of function. Almost half of participants reported needing to use their hands to push up to standing, which is an indicator of lower extremity weakness.

**Table 2:** Stay Independent Self-Assessment, Zuni Health Initiative, New Mexico, 2015

Stay Independent Checklist Question	Percentage of Yes Responses (N = 50)^a^
1. I have fallen in the past year.	52
2. I use or have been advised to use a cane or walker to get around safely.	36
3. Sometimes I feel unsteady when I am walking.	52
4. I steady myself by holding onto furniture when walking at home.	35
5. I am worried about falling.	71
6. I need to push with my hands to stand up from a chair.	48
7. I have some trouble stepping up onto a curb.	34
8. I often have to rush to the toilet.	31
9. I have lost some feeling in my feet.	16
10. I take medicine that sometimes makes me feel light-headed or more tired than usual.	16
11. I take medicine to help me sleep or improve my mood.	8
12. I often feel sad or depressed.	44

a There were only 49 responses for questions 4, 8, and 12.

Our second objective was to introduce the concept of modifiable fall risk factors as a first step to educating elders about personally relevant fall risk factors. The checklist survey stimulated an informal conversation following completion of the survey between the CHRs and the elders about falls and basic prevention strategies. The CHRs informally reported to the researchers that most elders expressed an interest in participating in a falls-prevention program if it was offered at Zuni Pueblo.

In meeting the third objective, a tribal advisory panel (TAP) was convened, and the outcomes of the study were presented to the group. The TAP has met twice to review a proposed study protocol and to provide feedback and guidance to the investigators regarding the conduct of a proposed study. Liaisons from existing tribal health programs have been identified and have agreed to participate as TAP members. Community coordinators were present at each of the health programs at Zuni and have agreed to attend the quarterly TAP. Importantly, if a falls prevention program proves successful in improving health outcomes, the tribe will enthusiastically attempt to sustain the program.

## Interpretation

The research team met with members from Zuni Senior Center, CHRs, and Zuni leadership (including the governor, council members, and Zuni IHS providers) to present the survey results including the high incidence of falls in their community. Fifty-two percent of study participants reported a fall within the past year. According to a newly published CDC report, in 2014, 28.7% of adults aged 65 years or older reported falling (19). The difference in reported falls highlights the need for effective intervention in this tribal community. To the best of our knowledge, Stay Independent self-assessment data have not been published for other populations, so we were unable to compare our data with those of older adults from other regions.

This community engagement project strengthened the established Zuni partnerships developed by the Zuni Health Initiative. It extended health promotion efforts from a diabetes education focus to education about elder falls risk and evidence-based interventions. Ultimately, we hope to implement an effective system for home-based, community-based, and IHS-based education, screening, and follow-up for falls prevention in the Zuni Pueblo with robust evaluation of these interventions.

The CDC Stay Independent checklist was accepted by the Zuni elder participants, easily administered by trained CHRs, and demonstrated an incidence of falls in a tribal community that was significantly higher than the national average for older adult falls. The Stay Independent checklist is a readily available resource, which engaged this tribal community to consider the issue of elder falls.

This collaboration was successful in that it engaged Zuni community members to evaluate culturally centered knowledge that contributed to awareness of the prevalence of falls in their elder community and ways of reducing fall risk. The Zuni Pueblo is an example of a collaborative effort to reduce health disparities in tribal communities and demonstrates a willingness in the community to pursue new initiatives (ie, falls prevention) through genuine and engaged partnerships.
